# Prospective evaluation of radiation‐induced skin toxicity in a race/ethnically diverse breast cancer population

**DOI:** 10.1002/cam4.608

**Published:** 2016-01-14

**Authors:** Jean L. Wright, Cristiane Takita, Isildinha M. Reis, Wei Zhao, Eunkyung Lee, Omar L. Nelson, Jennifer J. Hu

**Affiliations:** ^1^ Department of Radiation Oncology and Molecular Radiation SciencesJohns Hopkins UniversityBaltimoreMaryland; ^2^ Department of Radiation OncologyUniversity of Miami School of MedicineMiamiFlorida; ^3^ Department of Public Health SciencesUniversity of Miami School of MedicineMiamiFlorida; ^4^ Sylvester Comprehensive Cancer CenterUniversity of Miami School of MedicineMiamiFlorida

**Keywords:** Breast cancer, racial/ethnic disparity, radiation dermatitis, radiation therapy, skin toxicity

## Abstract

We evaluated predictors of radiation‐induced skin toxicity in a prospective study of a tri‐racial/ethnic breast cancer population. We evaluated patient demographics, tumor characteristics, and treatment variables in the first 392 patients in a prospective study assessing radiation‐induced skin toxicity. Logistic regression analyses were conducted to evaluate potential predictors of skin toxicity. The study consists of 59 non‐Hispanic whites (NHW; 15%), 241 Hispanic Whites (HW; 62%), 79 black or African Americans (AA; 20%), and 13 others (3%). Overall, 48% developed grade 0–1 skin toxicity, 49.8% grade 2, and 2.2% grade 3 by the National Cancer Institute's Common Toxicity Criteria for Adverse Events (CTCAE) scale. Twenty‐one percent developed moist desquamation. In multivariate analysis, higher body mass index (BMI; OR = 2.09; 95%CI = 1.15, 3.82), higher disease stage (OR = 1.82; 95%CI = 1.06, 3.11), ER‐positive/PR‐negative status (OR = 2.74; 95%CI = 1.26, 5.98), and conventionally fractionated regimens (OR = 3.25; 95%CI = 1.76, 6.01) were significantly associated with higher skin toxicity grade after adjustment for age, race, ethnicity, ER status, and breast volume. BMI specifically predicted for moist desquamation, but not degree of erythema. In this racially and ethnically diverse cohort of breast cancer patients receiving radiation to the intact breast, risk factors including BMI, disease stage, and conventionally fractionated radiation predicted for higher skin toxicity grade, whereas age, race, ethnicity, and breast volume did not. BMI specifically predicted for moist desquamation, suggesting that preventive measures to address this particular outcome should be investigated.

## Introduction

The majority of women with in situ and early‐stage breast cancer receive adjuvant breast radiation therapy (RT) after breast‐conserving surgery. Breast RT is generally well tolerated, but acute skin toxicity is a common side effect which can result in bothersome symptoms including burning sensation, itching, tenderness, and pain. In some cases, the skin reaction can progress to desquamation, either dry or moist, which is often more uncomfortable and poses a risk, albeit small, of infection and/or treatment breaks. Mild erythema is very common, occurring in up to 95% of patients, as is brisk erythema with or without moist desquamation, ranging from 5% to 69%, whereas moist desquamation is less common, ranging from 11% to 47% 1, 2, 3, 4, 5, 6, 7, 8. Several predictive factors for more severe skin toxicity have been identified, including body mass index (BMI), breast size, and radiation technique including fractionation regimen and dosimetric homogeneity 1, 4, 6, 7, 8, 9.

Our institution serves a racially and ethnically diverse population of breast cancer patients, and we have been interested in studying racial and ethnic variation in radiation‐related skin toxicity in breast cancer patients. We previously published a series evaluating predictors of skin toxicity in a cohort of patients receiving postmastectomy radiation (PMRT), and identified black/AA race, postmenopausal status, and higher BMI as predictors for moist desquamation 10. Interestingly, these same factors did not predict for higher grade skin toxicity by the National Cancer Institute's Common Toxicity Criteria for Adverse Events (CTCAE) skin toxicity scale, which does not clearly differentiate between patients who do and do not develop moist desquamation. The observed variation in skin toxicity in that series was thus seen primarily in rate of moist desquamation, rather than other skin toxicity characteristics such as the degree of erythema. Moist desquamation is much less common in patients receiving radiation to the intact breast after lumpectomy, as compared to patients receiving radiation to the chest wall after mastectomy. We therefore sought to determine if the same factors, particularly race, predicted for more severe skin toxicity in the setting of radiation to the intact breast in a similarly diverse cohort of patients from the same institution.

## Materials and Methods

In this study, we evaluated 392 consecutive breast cancer patients enrolled during December, 2008 to July, 2014 in a prospective study assessing RT‐induced skin toxicity to the intact breast in the Radiation Oncology Department at the University of Miami. The study was approved by the Institutional Review Board. Women (≥18 years) with newly diagnosed breast carcinoma, stage 0–III (American Joint Committee on Cancer) who underwent breast‐conserving surgery were scheduled to receive RT to the intact breast with or without regional nodal radiation were eligible. At the time of enrollment, patients signed informed consent either in English or Spanish, and completed a baseline assessment form, including self‐identification of race and ethnicity, breast cancer risk factors including reproductive and family history, as well as comorbidities, height, weight, and smoking habits. Other patient, disease, and treatment characteristics, including detailed information on radiation delivery, were prospectively collected.

Skin toxicity was assessed by the treating physician. As previously described 10, we used both NCI CTCAE (v3.0), and a modified variation in the NCI CTCAE (v3.0) skin toxicity scale which breaks CTCAE “grade 2” into three subcategories, seeking to capture more detailed information including the presence and extent of dry and moist desquamation. The scale divides skin reaction into six categories as follows: 1 – faint or dull erythema and/or follicular reaction and/or itching (CTCAE grade 1); 2 – bright erythema and/or tender to touch (CTCAE grade 2); 3 – dry desquamation with or without erythema (CTCAE grade 1 or 2); 4 – small or moderate amount of wet desquamation (CTCAE grade 2); 5 – confluent moist desquamation (CTCAE grade 3); 6 – ulceration, hemorrhage, and/or necrosis (CTCAE grade 4). Skin toxicity was captured at midpoint and at the completion of RT. In general, the duration of RT was 4 or 6 weeks depending on the fractionation scheme used. The patients in our cohort were uniformly managed with topical aloe vera applied to the breast throughout treatment, with silver sulfadiazine applied to areas of desquamation as needed.

We used Pearson's chi‐squared test or Fisher's exact test to compare differences in the distributions of patient and disease characteristics as well as skin toxicity grade by race/ethnicity. Wilcoxon signed‐rank test was performed to evaluate progression of RT‐induced skin toxicity from midpoint to RT completion. Multiple logistic regression analyses were conducted to evaluate the association between multiple predictors and the risk of higher grade skin toxicity using both grading scales. Statistical analysis was performed using SAS version 9.3 for Windows (SAS Institute, Cary, NC) and significance level was set at two‐sided *α* = 0.05.

## Results

### Patient demographic and tumor characteristics

In Table 1, we summarize overall patient, tumor, and treatment characteristics by race and ethnicity, presented as 15% non‐Hispanic white (NHW), 62% Hispanic white (HW), 20% AA, and 3% other, as well as condensed to 80% non‐AA and 20% AA. Mean age at the time of enrollment was 56.2 years (range 27–85 years). Thirty‐three percent were pre or perimenopausal, and 67% postmenopausal. A higher proportion of AA patients were obese (61% vs. 35% in non‐AA; *P* < 0.001), had at least two comorbidities (31% vs. 22%; *P* = 0.013), had stage II‐III disease (43% vs. 28%; *P* = 0.022), had ER‐negative tumors (34% vs. 21%; *P* = 0.013) or triple‐negative tumors (27% vs. 12%; *P* < 0.001), and had above‐median breast volume (72% vs. 45%; *P* < 0.001).

**Table 1:** Patient characteristics by race/ethnicity

Variable	Total		NHW		HW		AA		Other		*P* a	Non‐AA		AA		*P* a
	N	%	N	%	N	%	N	%	N	%		N	%	N	%	
Study population	392	100	59	15	241	62	79	20	13	3		313	80	79	20	
Age at consent (years)																
<50	98	25	18	30	58	24	20	25	2	15	0.776	78	25	20	25	0.733
50–59	156	40	21	36	94	39	34	43	7	54		122	39	34	43	
≥60	138	35	20	34	89	37	25	32	4	31		113	36	25	32	
Mean (SD)	56.2 (9.1)		55.9 (9.1)		56.5 (9.0)		55.5 (9.4)		57.9 (1.1)			56.4 (9.1)		55.5 (9.4)		
Menopausal status																
Pre/Peri	128	33	24	41	76	32	25	32	3	23	0.392	103	33	25	32	0.831
Post	264	67	35	59	165	68	54	68	10	77		210	67	54	68	
BMI (kg/m^2^)																
<25	101	26	28	47	55	23	13	16	5	38	**≤0.001**	88	28	13	16	**≤0.001**
25–29.99	133	34	17	29	94	39	18	23	4	31		115	37	18	23	
≥30	158	40	14	24	92	38	48	61	4	31		110	35	48	61	
Mean (SD)	29.4 (6.4)		27.0 (6.6)		29.0 (5.2)		32.5 (8.4)		28.6 (6.7)			28.6 (5.6)		32.5 (8.4)		
Smoking history																
Never	260	66	38	64	156	65	56	71	10	77	0.583	204	65	56	71	0.337
Ever	132	34	21	36	85	35	23	29	3	23		109	35	23	29	
Number of comorbidities^b^																
0	154	39	27	46	102	42	20	25	5	38	0.066	134	43	20	25	**0.013**
1	146	37	20	34	87	36	35	44	4	31		111	35	35	44	
2	66	17	7	12	36	15	20	26	3	23		46	15	20	26	
≥3	26	7	5	8	16	7	4	5	1	8		22	7	4	5	
Disease stage																
0	79	20	7	12	53	22	15	19	4	31	**0.013**	64	20	15	19	**0.022**
I	193	49	38	64	120	50	30	38	5	38		163	52	30	38	
II–III	120	31	14	24	68	28	34	43	4	31		86	28	34	43	
Histology																
DCIS (ductal carcinoma in situ)	85	22	8	14	57	24	16	20	4	31	0.692	69	22	16	20	0.954
IDC (invasive ductal carcinoma)	289	74	48	81	172	71	60	76	9	69		229	73	60	76	
ILC (invasive lobular carcinoma)	17	4	3	5	11	5	3	4	–	–		14	5	3	4	
Other	1	0	–	–	1	0	–	–	–	–		1	0	–	–	
ER																
Positive	299	76	43	73	193	80	52	66	11	85	**0.025**	247	79	52	66	**0.013**
Negative	92	24	16	27	47	20	27	34	2	15		65	21	27	34	
PR																
Positive	263	67	37	63	169	71	48	61	9	69	0.186	215	69	48	61	0.156
Negative	127	33	22	37	70	29	31	39	4	31		96	31	31	39	
HER2																
Positive	38	12	5	9	24	12	8	12	1	11	0.791	30	12	8	12	0.920
Negative	285	88	50	91	169	88	58	88	8	89		227	88	58	88	
Triple negative																
No	317	85	48	86	201	89	57	73	11	85	**0.003**	260	88	57	73	**≤0.001**
Yes	56	15	8	14	25	11	21	27	2	15		35	12	21	27	
Chemotherapy therapy																
No	191	49	28	48	119	49	41	52	3	23	0.869	150	48	41	52	0.306
Yes	201	51	31	52	122	51	38	48	10	77		163	52	38	48	
Hormone therapy																
No	133	34	17	29	75	31	40	51	1	8	**0.004**	93	30	40	51	**≤0.001**
Yes	257	66	42	71	165	69	39	49	11	92		218	70	39	49	
Fractionation																
Hypofractionated	67	17	12	21	41	17	12	15	2	15	0.698	55	18	12	15	0.560
Conventionally fractionated	323	83	46	79	199	83	67	85	11	85		256	82	67	85	
Lumpectomy cavity boost																
No	48	12	4	7	29	12	13	17	2	15	0.239	35	11	13	17	0.209
Yes	342	88	54	93	211	88	66	83	11	85		276	89	66	83	
Breast volume (cc)																
<881.3 (Median)	193	50	37	64	125	53	22	28	9	69	**≤0.001**	171	55	22	28	**≤0.001**
≥881.3	193	50	21	36	112	47	56	72	4	31		137	45	56	72	
Mean (SD)	999 (534)		820 (479)		976 (484)		1219 (638)		906 (583)			944 (990)		1219 (638)		
Percentage of breast volume with >105% prescription dose																
<51.3 (75th percentile)	261	75	38	69	159	76	57	79	7	58	0.419	204	74	57	79	0.337
≥51.3	88	25	17	31	51	24	15	21	5	42		73	26	15	21	
Mean (SD)	34.9 (24.8)		38.2 (26.1)		34.2 (24.6)		31.6 (24.5)		51.8 (17.5)			35.7 (24.9)		31.6 (24.5)		
Percentage of breast volume with >110% prescription dose																
0	184	53	28	51	114	54	39	54	3	25	0.889	145	52	39	54	0.805
>0	164	47	27	49	95	46	33	46	9	75		131	48	33	46	
Mean (SD)	16.1 (23.0)		24.3 (22.8)		13.5 (22.5)		16.1 (24.4)		18.8 (21.9)			16.1 (22.7)		16.1 (24.4)		

AA, Black or African American; HW, Hispanic white; NHW, non‐Hispanic white; SD, standard deviation; %, column percentage, except for table first row showing row percentage (distribution of study population by race/ethnicity).

a *P*‐value from chi‐squared test or Fisher's exact test excluding other race category and missing.

b Sum of 11 patient‐reported comorbidity conditions: diabetes, hypertension, heart disease, lung disease, thyroid condition, cirrhosis liver, stroke, chronic bronchitis, hepatitis, tuberculosis, etc.

Bold values indicate statistically significant findings at p < 0.05.

### Treatment characteristics

All patients received breast‐conserving surgery, and patients with invasive disease had axillary dissection or sentinel node biopsy. As shown in Table 1, 51% received systemic chemotherapy (8% neo‐adjuvant and 43% adjuvant) and 66% received hormone therapy. A higher proportion of AA patients did not receive hormone therapy (51% vs. 30%; *P* < 0.001). RT was delivered to the breast with or without regional nodes based on clinical indications. Patients were treated using standard or partially wide photon tangents with both conventionally fractionated and hypofractionated schemes. The dose range to the breast was 42.4–50.4 Gy, in fraction sizes of 1.8–2.7 Gy. The most common conventionally fractionated approach was 50 Gy in 2 Gy per fraction, and the most common hypofractionated approach was 42.4 Gy in 2.65 Gy per fraction. For the purposes of statistical analysis, total dose of <45 Gy in fraction size >2 Gy was considered hypofractionated, and total dose ≥45 Gy in fraction size of ≤2 Gy was considered conventionally fractionated.

Seventeen percent of patients were treated with a hypofractionated approach, and 83% with a conventionally fractionated approach. Regional nodal radiation including supra/infraclavicular nodes +/− axillary and intern al mammary nodes was delivered in 15% of patients, dose range 45–50.4 Gy in 25 fractions. Anterior oblique supraclavicular +/− axillary fields were most commonly matched monoisocentrically with the breast tangents. Eighty‐eight percent of patients received a boost to the lumpectomy cavity of 10–16 Gy. Planning was completed on the Eclipse or Pinnacle planning system depending on the institutional center, and forward planned field‐in‐field technique was used to maximize dose homogeneity. Dosimetric analysis showed that the mean percentage breast volume receiving >105% of prescription dose was 35% and >110% was 16%. There were no significant differences in RT treatment parameters by race or ethnicity.

### Skin toxicity

Table 2 demonstrates the progression of skin toxicity grades from midpoint to RT completion (*P* < 0.001). Using the modified grading scale, changes in skin toxicity from midpoint to RT completion were: (1) grade 0: decreased from 11% to 1%; (2) grade 1 (mild erythema): decreased from 82% to 42%; (3) grade 2 (brisk erythema without desquamation): increased from 4% to 20%; (4) grade 3 (dry desquamation with or without erythema): increased from 1% to 15%; (5) grade 4 (moist desquamation with or without erythema): increased from 3% to 20%; (6) grade 5 (confluent moist desquamation): increased from 0% to 2%. Using the NCI CTCAE grading scale, changes in skin toxicity from midpoint to RT completion were as follows: (1) grade 0: decreased from 11% to 1%; (2) grade 1: decreased from 83% to 46%; (3) grade 2: increased from 6% to 51%; and (4) grade 3: increased from 0% to 2%. No patient developed grade 6 by the modified study scale or grade 4 or greater by CTCAE scale at RT completion.

**Table 2:** Progression of skin toxicity from RT midpoint to post‐RT

Skin toxicity at RT midpoint (modified grade)	Skin toxicity at post‐RT (modified grade)							
	0	1	2	3	4	5	Total	*P* a
0	2	19	5	11	5	1	43 (11%)	**≤0.001**
1	1	140	63	43	58	7	312 (82%)	
2	–	1	6	3	3	–	13 (4%)	
3	–	–	–	1	2	–	3 (1%)	
4	–	–	2	–	6	1	9 (3%)	
Total	3 (1%)	160 (42%)	76 (20%)	58 (15%)	74 (20%)	9 (2%)	380	

Skin toxicity at RT midpoint (CTCAE grade)	Skin toxicity at post‐RT (CTCAE grade)							
	0	1	2	3	–	–	Total	*P* a
0	2	24	16	1	–	–	43 (11%)	**≤0.001**
1	1	149	157	7	–	–	314 (83%)	
2	–	1	21	1	–	–	23 (6%)	
Total	3 (1%)	174 (46%)	194 (51%)	9 (2%)	–	–	380	

a Wilcoxon signed‐rank test.

Bold values indicate statistically significant findings at p < 0.05.

Table 3 presents skin toxicity at RT completion using both the modified scale of 0–6, as well as the NCI CTCAE scale of 0–4, broken down by patient, disease, and treatment characteristics. In general, the two scales identified similar predictors of more severe skin toxicity, including higher BMI, more advance tumor stage and invasive ductal histology, progesterone receptor (PR) negative status, conventionally fractionated regimens with RT dose to whole breast ≥45 Gy, the use of a lumpectomy cavity boost, and above‐median breast volume. Breast volume and BMI were significantly correlated. Neither race nor ethnicity predicted for more severe skin toxicity grade, although there was a higher crude rate of severe skin reaction in AA patients compared to non‐AA: 28 versus 19% for modified grade 4–5 (moist desquamation) and 58 versus 50% for CTCAE grade 2–3 toxicity. Skin toxicity grade did not vary with age, menopausal status, the use of chemotherapy, and dosimetric factors.

**Table 3:** Skin toxicity grade at post‐RT by patient and clinical variables

	Skin toxicity (modified grade)														Skin toxicity (CTCAE grade)									
	0	1	2	3	4	5	*P* a	0–1		2–3		4–5		*P* b	0	1	2	3		0–1		2–3		*P* b
Variable	*N*	*N*	*N*	*N*	*N*	*N*		*N*	%	*N*	%	*N*	%		*N*	*N*	*N*	*N*	*P* a	*N*	%	*N*	%	
Total patients	4	169	77	59	74	9		173	44	136	35	83	21		4	184	195	9		188	48	204	52	
Age at consent (years)																								
<50	–	39	15	18	24	2	0.221	39	40	33	34	26	27	0.367	–	43	53	2	0.308	43	44	55	56	0.332
50–59	1	65	37	23	24	6		66	42	60	38	30	19		1	71	78	6		72	46	84	54	
≥60	3	65	25	18	26	1		68	49	43	31	27	20		3	70	64	1		73	53	65	47	
Menopausal status																								
Pre/Peri	–	56	19	23	27	3	0.433	56	44	42	33	30	23	0.719	–	61	64	3	0.996	61	48	67	52	0.933
Post	4	113	58	36	47	6		117	44	94	36	53	20		4	123	131	6		127	48	137	52	
Race/ethnicity																								
NHW	–	28	17	7	6	1	NE	28	47	24	41	7	12	0.451	–	31	27	1	0.286	31	53	28	47	0.610
HW	2	107	41	40	48	3		109	45	81	34	51	21		2	116	120	3		118	49	123	51	
AA	2	28	16	11	17	5		30	38	27	34	22	28		2	31	41	5		33	42	46	58	
Other	–	6	3	1	3	–		6	46	4	31	3	23		–	6	7	–		6	46	7	54	
Non‐AA	2	141	61	48	57	4	0.080	143	46	109	35	61	19	0.230	2	153	154	4	**0.019**	155	50	158	50	0.218
AA	2	28	16	11	17	5		30	38	27	34	22	28		2	31	41	5		33	42	46	58	
BMI (kg/m^2^)																								
<25	‐	56	18	15	10	2	**0.008**	56	55	33	33	12	12	**0.001**	‐	62	37	2	**0.004**	62	61	39	39	**0.002**
25–29.99	1	62	28	20	21	1		63	47	48	36	22	17		1	63	68	1		64	48	69	52	
≥30	3	51	31	24	43	6		54	34	55	35	49	31		3	59	90	6		62	39	96	61	
Smoking history																								
Never	2	111	45	39	56	7	0.227	113	43	84	32	63	24	0.093	2	121	130	7	0.738	123	47	137	53	0.717
Ever	2	58	32	20	18	2		60	45	52	39	20	15		2	63	65	2		65	49	67	51	
No. of comorbidities																								
None	1	67	34	25	24	3	NE	68	44	59	38	27	18	0.821	1	72	78	3	0.642	73	47	81	53	0.993
1	–	66	27	20	31	2		66	45	47	32	33	23		–	71	73	2		71	49	75	51	
2	3	25	10	12	14	2		28	42	22	33	16	24		3	29	32	2		32	48	34	52	
≥3	–	11	6	2	5	2		11	42	8	31	7	27		–	12	12	2		12	46	14	54	
Disease stage																								
0	2	45	11	10	10	1	0.099	47	59	21	27	11	14	**0.013**	2	47	29	1	**0.022**	49	62	30	38	**0.005**
IA–B	1	83	35	29	40	5		84	44	64	33	45	23		1	92	95	5		93	48	100	52	
IIA–IIIC	1	41	31	20	24	3		42	35	51	43	27	23		1	45	71	3		46	38	74	62	
Histology																								
DCIS (ductal carcinoma in situ)	2	49	10	11	11	2	NE	51	60	21	25	13	15	**0.004**	2	51	30	2	NE	53	62	32	38	**0.002**
IDC (invasive ductal carcinoma)	2	108	64	47	61	7		110	38	111	38	68	24		2	121	159	7		123	43	166	57	
ILC (invasive lobular carcinoma)	–	11	3	1	2	–		11	65	4	24	2	12		–	11	6	–		11	65	6	35	
Other	–	1	–	–	–	–		1	100	–	–	–	–		–	1	–	–		1	100	–	–	
ER																								
Positive	3	133	54	46	55	8	0.493	136	45	100	33	63	21	0.518	3	145	143	8	0.275	148	49	151	51	0.233
Negative	1	35	23	13	19	1		36	39	36	39	20	22		1	38	52	1		39	42	53	58	
PR																								
Positive	3	123	46	38	46	7	0.150	126	48	84	32	53	20	0.063	3	133	120	7	**0.044**	136	52	127	48	**0.022**
Negative	1	44	31	21	28	2		45	35	52	41	30	24		1	49	75	2		50	39	77	61	
HER2																								
Positive	–	17	7	6	8	–	0.836	17	45	13	34	8	21	0.855	–	19	19	–	0.503	19	50	19	50	0.500
Negative	2	112	62	43	58	8		114	40	105	37	66	23		2	124	151	8		126	44	159	56	
Triple negative																								
No	3	140	60	48	58	8	0.336	143	45	108	34	66	21	0.192	3	153	153	8	0.145	156	49	161	51	0.062
Yes	–	18	15	8	14	1		18	32	23	41	15	27		–	20	35	1		20	36	36	64	
Chemotherapy																								
No	3	86	37	27	34	4	0.909	89	47	64	34	38	20	0.622	3	91	93	4	0.604	94	49	97	51	0.628
Yes	1	83	40	32	40	5		84	42	72	36	45	22		1	93	102	5		94	47	107	53	
Hormone therapy																								
No	–	56	27	20	27	3	0.966	56	42	47	35	30	23	0.784	–	60	70	3	0.668	60	45	73	55	0.379
Yes	4	113	49	39	46	6		117	46	88	34	52	20		4	124	123	6		128	50	129	50	
Fractionation																								
Hypofractionated	2	46	10	4	5	–	**≤.001**	48	72	14	21	5	7	**≤.001**	2	47	18	–	**≤.001**	49	73	18	27	**≤.001**
Conventionally fractionated	2	122	67	54	69	9		124	38	121	37	78	24		2	135	177	9		137	42	186	58	
Lumpectomy cavity boost																								
No	1	30	5	5	5	2	**0.024**	31	65	10	21	7	15	**0.009**	1	31	14	2	**0.008**	32	67	16	33	**0.005**
Yes	3	138	72	53	69	7		141	41	125	37	76	22		3	151	181	7		154	45	188	55	
Breast volume																								
<881.3 CC (median)	4	94	34	30	27	1	**0.005**	99	51	66	34	28	15	**0.003**	4	100	85	1	**0.003**	105	54	88	46	**0.011**
≥881.3 CC	–	73	42	28	43	8		73	38	67	35	53	27		–	80	106	8		80	41	113	59	
Percentage of breast volume with >105% prescription dose																								
<51.3 (75th percentile)	3	118	48	42	45	5	0.607	121	46	90	34	50	19	0.438	3	126	127	5	0.581	129	49	132	51	0.644
≥51.3	1	43	15	9	19	1		44	50	24	27	20	23		1	45	41	1		46	52	42	48	
Percentage of breast volume with >110% prescription dose																								
0	2	89	29	29	33	2	0.634	91	49	58	32	35	19	0.710	2	94	86	2	0.350	96	52	88	48	0.456
>0	2	72	33	22	31	4		74	45	55	34	35	21		2	77	81	4		79	48	85	52	

NE, not estimable.

a *P*‐value from chi‐squared test or Fisher's exact test with grade 0–1 grouped.

b *P*‐value from chi‐squared test or Fisher's exact test.

Bold values indicate statistically significant findings at p < 0.05.

As shown in Table 4, multivariate analyses were performed to evaluate the association between skin toxicity and age, race, breast volume, BMI, stage, ER and PR status, fractionation approach, and breast volume. For the modified scale, analysis was performed for two separate groupings, grade 2–3 versus 0–1, and 4–5 versus 0–1; the first grouping separates patients with lower versus higher degrees of erythema or hyperpigmentation, whereas the latter specifically separates out patients with moist desquamation. For grade 2–3 versus 0–1, the following factors were significant: higher stage (OR = 1.82, 95% CI = 1.00, 3.31), ER‐positive/PR‐negative status (OR = 3.00 95% CI = 1.25, 7.21), and conventionally fractionated regimens (OR = 2.98; 95% CI = 1.52, 5.84); higher BMI was not significantly associated with higher grade toxicity. For 4–5 versus 0–1, higher BMI (OR = 2.99, 95% CI = 1.29, 6.92), ER‐positive/PR‐negative status (OR = 3.50, 95% CI = 1.29, 9.48), and conventionally fractionated regimens (OR = 4.81, 95% CI = 1.77, 13.05) were significantly associated with higher grade skin toxicity‐ specifically moist desquamation.

**Table 4:** Associations between multiple variables and post‐RT skin toxicity

Variable	Category	Skin toxicity (modified grade)				Skin toxicity (CTCAE Grade)	
		Model 1				Model 2	
		2–3 versus 0–1		4–5^a^ versus 0–1		2–3 versus 0–1	
		OR (95%CI)	*P*	OR (95%CI)	*P*	OR (95%CI)	*P*
Age at enrollment (years)	<50 versus ≥50	1.02 (0.57, 1.81)	0.948	1.44 (0.75, 2.75)	0.276	1.12 (0.67, 1.86)	0.670
Race	AA versus Non‐AA	0.95 (0.50, 1.78)	0.861	1.13 (0.56, 2.29)	0.735	1.01 (0.58, 1.76)	0.975
BMI	25–29.99 versus <25	1.32 (0.71, 2.47)	0.378	1.62 (0.70, 3.77)	0.262	1.84 (1.03, 3.27)	**0.038**
	≥30 versus <25	1.53 (0.79, 2.98)	0.210	2.99 (1.29, 6.92)	**0.011**	2.09 (1.15, 3.82)	**0.016**
Stage	I–III versus 0	1.82 (1.00, 3.31)	**0.050**	2.10 (0.98, 4.50)	0.058	1.82 (1.06, 3.11)	**0.029**
ER/PR	ER+/PR− versus ER+/PR+	3.00 (1.25, 7.21)	**0.014**	3.50 (1.29, 9.48)	**0.014**	2.74 (1.26, 5.98)	**0.001**
	ER−/PR− versus ER+/PR+	1.66 (0.93, 2.97)	0.087	1.45 (0.72, 2.96)	0.300	1.57 (0.93, 2.66)	0.095
Fractionation	Conventional versus Hypo	2.98 (1.52, 5.84)	**0.001**	4.81 (1.77, 13.05)	**0.002**	3.25 (1.76, 6.01)	**≤.001**
Breast volume (CC)	≥Median versus <Median	1.29 (0.74, 2.23)	0.371	1.87 (0.96, 3.63)	0.067	1.38 (0.84, 2.27)	0.200

OR: odds ratio; CI: confidence interval; Model 1: multinomial logistic regression with generalized logit link function. Model 2: logistic regression.

a Presence of moist desquamation.

Bold values indicate statistically significant findings at p < 0.05.

Using the NCI CTCAE grading scale, higher BMI (OR = 2.09; 95% CI = 1.15, 3.82), higher stage (OR = 1.82; 95% CI = 1.06, 3.11), ER‐positive/PR‐negative status (OR = 2.74; 95% CI = 1.26, 5.98), and conventionally fractionated regimens (OR = 3.25; 95% CI = 1.76, 6.01) were significantly associated with higher grade RT‐induced skin toxicity (2–3 vs. 0–1). After controlling for all predictors, age, race, and breast volume were not significant predictors of severe skin toxicity by either grading scale.

## Discussion

In this prospectively followed tri‐racial/ethnic cohort of breast cancer patients receiving adjuvant RT to the intact breast after breast‐conserving surgery, the overall incidence of NCI CTCAE grade 2 or greater skin toxicity was 52%, and 21% developed moist desquamation, consistent with the majority of published series 1, 2, 3, 4, 5, 6, 7, 8. We identified higher BMI, higher disease stage, PR‐negative tumor status, and conventionally fractionated regimens as predictors for higher skin toxicity grade. Age, race, ethnicity, and breast volume did not predict for skin toxicity. Additionally, a more detailed skin toxicity scale designed to specifically capture desquamation identified BMI as a predictor specifically for moist desquamation, but not dry desquamation or degree of erythema, a finding the NCI CTCAE scale was not able to detect.

One of the most important findings of this series is that race and ethnicity were not associated with variation in skin toxicity. In our previously published series of patients receiving PMRT, the incidence of moist desquamation was much higher, 53.7% overall, and AA race was found to be a significant predictor of moist desquamation but not of higher grade skin toxicity by the CTCAE scale 10. In the current series, there was a nonsignificantly higher rate of severe skin toxicity in AA versus non‐AA patients – 58 versus 50% for CTCAE grade 2–3 toxicity, and 28 versus 19% for moist desquamation. AA patients were more likely to have other potential risk factors for skin toxicity, including higher BMI, higher disease stage, and larger breast volume; when these factors were considered in multivariate analysis, race ultimately did not predict for skin toxicity grade or moist desquamation. There are two possible interpretations. One is that this study has limited statistical power to identify variation in skin toxicity by race, given the low incidence of moist desquamation and severe skin toxicity in patients receiving radiation to the intact breast. However, it is also possible that race only predicts for moist desquamation at the higher skin doses achieved using PMRT, and is not associated with skin toxicity grade in the postlumpectomy setting. We look forward to analysis of additional cohorts to determine if differences are seen with larger patient numbers and different populations.

It is also important to note that this cohort includes a large number of HW patients, who make up the majority (62%) of the non‐AA comparison group, demonstrating no increased risk of skin toxicity severity in this population as compared to NHW and AA patients.

The relationship between BMI and higher grade skin toxicity is supported by previous studies 6, 8, 9. However, our findings on multivariate analysis using the modified scale additionally demonstrated that BMI is specifically associated with moist desquamation, rather than dry desquamation or greater degree of erythema or hyperpigmentation. While both breast volume and BMI predicted for higher skin toxicity grade in univariate analysis, only BMI retained statistical significance on multivariate analysis, suggesting this is a more important predictor than breast volume. This finding likely relates the bolus effect of skin folds seen in obese patients, as well as the abrasive effect of friction within skin folds; nonobese patients with larger breasts often have fewer skin folds than obese patients, explaining why BMI may be more predictive than breast volume. Skin toxicity is usually addressed with one of any number of topical agents, or in some cases with subcutaneous amifostine, 6, 11, 12, but recent data suggest that a protective barrier approach may also reduce desquamation 13. The premise of the barrier film approach is that skin reaction forms from an accumulation of microabrasions on the skin surface, in tissue that is sensitized to injury by radiation. The finding that BMI is specifically correlated with moist desquamation points to a barrier approach as a potentially more effective approach in these patients, an important subject for future investigation.

Large breast separation (a surrogate for large breast volume and/or BMI) has long been considered a relative contraindication to hypofractionated treatment regimens, based on the concept that such patients are at higher risk of more severe skin reaction. One of the reasons for the risk of skin toxicity in patients with large breast separation has been the difficulty in achieving dose homogeneity in this setting, and the awareness that dosimetric “hotspots” are likely to increase the risk of desquamation 1. However, this series demonstrates that relatively homogeneous plans can be achieved even in patients with large breast volume and/or high BMI. About 40% of our patients were obese. Nonetheless, the mean percent of the breast volume receiving >105% of prescription dose was 35%, and >110% was 16%. Dosimetric factors were not associated with skin toxicity, possibly because reasonably homogeneous plans were achieved. The finding that higher BMI predicted for moist desquamation, whereas dosimetric factors did not, again suggests skin folds as an important underlying cause of moist desquamation.

The majority of data evaluating toxicity related to fractionation scheme has focused on late rather than acute toxicity. However, a recent large analysis from the Michigan Radiation Oncology Quality Consortium found that conventionally fractionated radiation was associated with higher skin toxicity grade compared to hypofractionated regimens 8, and our study corroborates this finding. The reasons for this likely relate to the lower total dose prescribed with hypofractionated regimens, and this finding lends greater support for the use of this approach in appropriately selected patients 8, 14.

There are a number of interesting findings in our analysis, in particular associations between skin toxicity and disease characteristics including stage and receptor status. It is interesting that PR‐negative status predicted for higher skin toxicity grade in this series, whereas in our series evaluating risk factors for skin toxicity in the setting of PMRT, PR‐negative status was protective. There is no clear explanation for these findings; we are not aware of any literature that identifies hormone receptors as a predictor of RT‐induced skin reaction 15. The fact that PR status emerged as a significant predictor in both series, but in opposite directions, suggests that it is possible that these relationships are treatment specific or related to the statistical limitations of these relatively small series. To better evaluate the relationship identified in this series, we conducted additional analyses and found that ER and PR status were significantly associated with each other on univariate analysis (data not shown); thus in the multivariate model we included ER and PR status as a combined variable, to avoid collinearity. PR status was analyzed as ER+/PR− versus ER+/PR+, and as ER−/PR− versus ER+/PR+ to account for this, and in the context of ER positivity, PR‐negative status retained its significance as a risk factor for more severe skin toxicity. Thus, it seems the strength of this relationship is maintained despite the collinearity of ER and PR. These relationships between PR status and skin toxicity may thus be a novel finding, which requires further investigation.

Our analysis also showed that more severe skin toxicity was associated with invasive disease as compared to DCIS, but not chemotherapy or hormone therapy. There may be underlying changes in the skin of the breast in the setting of invasive breast cancer or more locally advanced breast cancer that predispose to radiation sensitivity. It would seem logical that the skin toxicity grade would relate to the more frequent use of chemotherapy in patients with invasive disease, but there have been mixed findings on this correlation 16, 17, and these associations may also relate to the time interval between chemotherapy and radiation.

Overall this series identified a number of factors that were associated with skin reaction that are not readily explained, including relationships between tumor subtype, stage, and skin reaction. While there is no known mechanism for such relationships, we hypothesize that these findings may relate to patient factors such as inflammatory or other cytokines related to the various conditions – obesity, presence of invasive breast cancer, and PR status, among others, that might link these factors to skin toxicity. Our prospective analysis also includes collection of genomic DNA, serum, and urine specimens at the start and completion of RT, and we are optimistic that future studies may begin to elucidate molecular and genetic mechanisms 18. Indeed, our preliminary analysis has uncovered a relationship between C‐reactive protein and skin reaction 19. However, the relationships identified in this study must be interpreted cautiously, and are simply hypothesis‐generating at this time.

In this series, the NCI CTCAE scale captured the majority of the findings that the modified scale did, however, the more nuanced analysis of the study scale was able to differentiate between factors that increased the risk of higher grade skin toxicity overall (including erythema, hyperpigmentation, and desquamation), as well as factors that specifically predict for moist desquamation. These findings lend additional support to the need to capture additional skin toxicity data beyond the CTCAE scale.

We continue to expand this study cohort and will conduct additional analyses as our data matures. With a rate of moist desquamation of 21%, we hope that we may be able to strengthen the statistical analysis and more clearly identify novel predictors of this endpoint as our series continues to grow over time. As a component of our study we are also collecting patient‐reported outcomes in the form of the Breast Cancer Treatment Outcome Scale (BC‐TOS) and we are currently conducting an analysis of quality of life (QOL) data in this patient cohort, relating QOL outcomes to acute skin toxicity factors, to help put the acute toxicities identified in this study in context of the patient‐reported outcomes and to guide priorities for future treatment decision making and intervention studies.

## Conflict of Interest

None declared.
